# Analysis and prediction of readmissions for heart failure in the first year after discharge with INCA score

**DOI:** 10.1038/s41598-023-49390-w

**Published:** 2023-12-18

**Authors:** Alessia Rubini, Cristina Vilaplana-Prieto, Elena Vázquez-Jarén, Miriam Hernández-González, Francisco Javier Félix-Redondo, Daniel Fernández-Bergés

**Affiliations:** 1 PhD Programme in Economics (DEcIDE), International Doctorate School of the National University of Distance Education (EIDUNED), 28015 Madrid, Spain; 2 Research Unit of Don Benito-Villanueva de la Serena Health Area, 06700 Villanueva de la Serena, Spain; 3 Faculty of Economics and Business, University of Murcia, 30100 Murcia, Spain; 4 University Institute for Biosanitary Research of Extremadura (INUBE), 06080 Badajoz, Spain; 5 Villanueva Norte Health Centre, Extremadura Health Service, 06700 Villanueva de la Serena, Spain

**Keywords:** Health care economics, Quality of life

## Abstract

To determine the readmissions trends and the comorbidities of patients with heart failure that most influence hospital readmission rates. Heart failure (HF) is one of the most prevalent health problems as it causes loss of quality of life and increased health-care costs. Its prevalence increases with age and is a major cause of re-hospitalisation within 30 days after discharge. INCA study had observational and ambispective design, including 4,959 patients from 2000 to 2019, with main diagnosis of HF in Extremadura (Spain). The variables examined were collected from discharge reports. To develop the readmission index, capable of discriminating the population with higher probability of re-hospitalisation, a Competing-risk model was generated. Readmission rate have increased over the period under investigation. The main predictors of readmission were: age, diabetes mellitus, presence of neoplasia, HF without previous hospitalisation, atrial fibrillation, anaemia, previous myocardial infarction, obstructive pulmonary disease (COPD) and chronic kidney disease (CKD). These variables were assigned values with balanced weights, our INCA index showed that the population with values greater than 2 for men and women were more likely to be re-admitted. Previous HF without hospital admission, CKD, and COPD appear to have the greatest effect on readmission. Our index allowed us to identify patients with different risks of readmission.

## Introduction

Heart failure (HF) is one of the most prevalent health problems in developed countries. Worldwide, it is estimated that 64.3 million people suffer from HF^1^, with an approximate prevalence between 1 and 2% of the adult population^2,3^. Despite improvements in the management and treatment of HF in recent years, the percentage of readmissions continues to be high. Currently, HF remains the disease with the highest 30-day readmission rate^4^.

The prevalence of HF increases with age^5^. Taking into account that the life expectancy of the global population is increasing and that patients with HF are more susceptible to recurrent hospitalization due to decompensation of their chronic pathologies, the increase in the number of readmissions in recent years^6,7^ takes on special importance. In a public health context, in addition to the associated reduced quality of life^8^, it is essential to consider the costs generated by hospital readmissions^9,10^. Focusing on economic terms, HF generates a considerable burden to the health care system through the combined costs of admission, readmission, nursing staff, and medications. According to a study based in Spain^11^, the average total annual cost of HF ranges from 12,995 euros to 18,220 euros, depending on the level of severity of the disease and the comorbidities present.

Considering this, methodologically comprehensive studies that can describe the readmission phenomenon as a whole, including an analysis of the presence of comorbidities, are needed. To respond to this need, we aimed to determine the readmissions trends over the years and the comorbidities of patients with HF that most influence hospital readmission rates within one year of discharge by means of an indicator capable of analysing specific factors.

## Materials and methods

The INCA study^12^ has an observational and ambispective design. A total of 4,959 individuals, representing all patients with a primary diagnosis of HF at discharge (428 International Classification of Diseases 9th Revision [ICD-9] codes for the period 2000–2015 along with i50.0, i50.1, and i50.9 from the 10th Revision [ICD-10]) during the period 2000–2019 admitted consecutively to the hospital complex of the Don Benito-Villanueva de la Serena Health Area (which initially consisted only of the Don Benito-Villanueva Hospital and, since 2008, also the Siberia-Serena Hospital in Talarrubias), were included. This Health Area has a population of 141,337 inhabitants.

The data were obtained through the Coding and Computing Service of the Hospital Don Benito-Villanueva and the Subdirectorate of Information of the Extremadura Health Service. The information on mortality was obtained by contrasting the hospital base with the National Death Index (INDeF). The ethics committee of the University Hospital of Badajoz approved this study, that was conducted in accordance with the relevant guidelines and regulations.

### Variables

The following variables were collected from each of the discharge reports: sex; age; length of stay; presence of peripheral arterial disease (PAD); previous stroke; previous myocardial infarction(MIp); history of HF without hospital admission; comorbidities (neoplasms and chronic obstructive pulmonary disease [COPD] defined as the presence of COPD and/or obstructive sleep apnoea [OSA]); and cardiovascular risk factors (arterial hypertension, diabetes mellitus [DM], and atrial fibrillation [AF]).

Laboratory test results were also consulted. To screen for chronic kidney disease (CKD), the glomerular filtration rate was obtained using the CKD-EPI formula^13^ with a cut-off of < 45 ml/min/m^3^. Anaemia^14^ was screened for using the following haemoglobin cut-offs: < 13 g/dL for men and < 12 g/dL for women.

Hospital readmissions for HF in the first year after discharge were then analysed.

All investigators who participated in the study were trained at the study coordinating centre to ensure the quality of the data.

### Statistical analysis

Continuous variables are presented as the mean and standard deviation or median and interquartile range, while categorical variables are presented as the absolute and relative frequency. Differences between the living and the dead were compared using the chi-square test. To construct the hospital readmission, index a Cox proportional hazards model, and then, a competing-risk model^15,16^ (special type of survival analysis that aims to correctly estimate the marginal probability of an event—readmission- in the presence of competing events—death during the 1 years after discharge) were generated. The patients who died during hospital admission were excluded and only patients discharged alive were assessed.

Although our primary hypotheses concern non-fatal endpoints, mortality is a competing risk in this population. Therefore, significant variables were selected for the composite endpoint of death or readmission in the 1-year post-discharge period.

The model was adjusted for age and only those variables that had an unadjusted association with the occurrence of hospital readmission for HF and a *p* value < 0.05 were considered for inclusion (i.e., length of hospital stay, DM, neoplasia, MIp, previous HF, moderate valvulopathy, anaemia, COPD, OSAS, CKD).

All analyses were bilateral, and 95% confidence intervals (CIs) were calculated using the bootstrapping analysis with 1,000 replications to ensure model robustness, with IBM SPSS and STATA version 17.

### Construction of the indicator

To construct a score and an indicator capable of predicting the risk of readmission due to HF separated by sex, the variables were weighted differently according to the value of the hazard ratio (hazard ratio up to 0.50: 0.5 points, hazard ratio between 0.51 and 1: 1 point). Presence of DM, neoplasia, AF, anaemia and MIp score 0.5 points, while previous HP, CKD and COPD/OSAS score 1 point.

The model was then adjusted for age (> 75 years: 0.5 points, < 75 years: 0 points.

### Ethics approval

Approval was obtained from the ethics committee of University Hospital of Badajoz.

### Consent to participate

Informed consent was obtained from legal guardians.

## Results

A total of 4,959 patients admitted between January 1, 2000 and December 31, 2019 were included in the INCA study. Those who died in hospital readmissions (*n* = 473) were excluded, leaving 4,486 patients who were followed up. The average age was 77.21 (± 10.40), and 53.8% (*n* = 2,414) were women.

The overall readmission rate of patients with a diagnosis of HF in the first year after discharge was 27.3%. Table 1 shows the increase in the readmission rate over the 20-year follow-up period grouped into 5-year periods. An upward trend in the rate of readmissions, especially 30-day readmissions, were seen. During the period from 2015 to 2019, the rate of readmissions had doubled the initial percentage.

**Table 1:** Readmissions at one month, at 6 months and at one year, in groups of 5 years, from 2000 to 2019.

Groups by 5 years		Frequency	% of readmissions over the 5-year readmissions total	% of readmissions over the 20-year readmissions total
2000–2004	from 0 to 30 days	28	17.1%	2.3%
	from 31 to 180 days	77	47.0%	6.3%
	from 181 to 365 days	59	36.0%	4.8%
	Total of 2000–2004	164	100.0%	13.4%
2005–2009	from 0 to 30 days	49	24.3%	4.0%
	from 31 to 180 days	91	45.0%	7.4%
	from 181 to 365 days	62	30.7%	5.1%
	Total of 2005–2009	202	100.0%	16.5%
2010–2014	from 0 to 30 days	63	26.1%	5.2%
	from 31 to 180 days	128	53.1%	10.5%
	from 181 to 365 days	50	20.7%	4.1%
	Total of 2010–2014	241	100.0%	19.7%
2015–2019	from 0 to 30 days	213	34.6%	17.4%
	from 31 to 180 days	269	43.7%	22.0%
	from 181 to 365 days	134	21.8%	11.0%
	Total of 2015–2019	616	100.0%	50.4%
	Total of 2000–2019	1,223	**-**	100.0%

The results of the Competing-risk model are presented in Table 2. The following variables were predictors of readmission in the first year: age, DM, presence of neoplasms, HF without hospital admission, AF, anaemia, MIp, COPD, CKD.

**Table 2:** Competing risk analysis for the readmission study, with death before the 1 year after discharge as a competing risk.

Variables	SHR	Robust Std. Err	P >|z|	[95% Conf. Interval]	
Age	1.0219	.0035	0.000	1.0150	1.0289
DM	1.2010	.0710	0.002	1.0696	1.3487
Neoplasia	1.3185	.1080	0.001	1.1229	1.5480
Previous HF	1.7633	.1070	0.000	1.5656	1.9859
AF	1.2291	.0730	0.001	1.0941	1.3808
Anaemia	1.3129	.0826	0.000	1.1606	1.4853
MIp	1.3175	.1071	0.001	1.1235	1.5450
COPD-OSAS	1.7147	.2385	0.000	1.3055	2.2522
CKDEPI 45 ml/min	1.5257	.0954	0.000	1.3496	1.7247

Then, was constructed the INCA score using the values and procedures specified in the methodology section. Based on the scores of these 4,486 study participants (Table 3), the threshold represented by 2 points in men and women appears to be where the percentage of participants readmitted is consistently higher than that of participants who are not readmitted, thus creating two distinct populations with a different probability of readmission. Pearson’s chi-square significance was determined to be 0.001.

**Table 3:** Points obtained in the INCA score, divided into women and men.

Men	Percentage of patients NOT readmitted	Percentage of patients readmitted	Women	Percentage of patients NOT readmitted	Percentage of patients readmitted
Points on the index			Points on the index		
0,0	**3,6**	1,4	0,0	**3,1**	0,9
0,5	**10,8**	3,2	0,5	**11,4**	5,3
1,0	**18,4**	10,4	1,0	**17,6**	9,9
1,5	**18**	16	1,5	**16,1**	12,8
2,0	**16,2**	13,7	2,0	**17,4**	15,9
2,5	13,8	**23,4**	2,5	13,6	**20,2**
3,0	8,2	**12**	3,0	9,9	**15,6**
3,5	4,1	**9**	3,5	4,9	**9,6**
4,0	2,8	**6,2**	4,0	2,3	**6,1**
4,5	0,8	**2,5**	4,5	0,2	**1,5**
5,0	0,1	**0,5**	5,0	-	**0,3**

Significant values are in [bold].

## Discussion

### Main findings

Our study shows that the readmission rate of patients with HF increased steadily over this 20-year period, with the greatest burden in the first 30 days after discharge.

Predictors of readmission for both sexes were as follows: a history of CKD, COPD, and previous HF without hospitalization. We propose a risk score that can facilitate the identification of patients who are likely to suffer from it.

Since our study consecutively included patients with a diagnosis of decompensated HF over a 20-year period, the results are considered representative of the study area. The INCA study is comparable with other international “real world” studies^17,18^ that differ from clinical trials^19,20^ and exclusive registries from cardiology services^21,22^ in that they register at least 50% women, include populations that are older, and those with a high rate of comorbidity.

#### Analysis of the population and readmissions at one month, six months, and one year

Table 4 shows the percentage of readmissions at 30 days, six months, and one year reported in the INCA study and other international studies. To date, the need for a clear separation by risk of readmissions, according to these three periods, has not been addressed. The information provided in this study suggests that readmissions at one month and one year may be motivated by different causes. While the former is usually related to treatment imbalances or early discharge, readmission after six months or one year are usually due to poor adherence to treatment^19^, lack of follow-up after discharge, or the natural progression and severity of the disease.

**Table 4:** Comparison between INCA and other studies of the population, gender, and readmission rates at 30 days, at 6 months and one year.

	INCAex	Martinez-Santos (2019)	Giakoumis (2020)	Wideqvist (2021)	Omersa (2016)
N	4.959	77.652	51.141	448	34.406
Female (%)	53,8	55,3	55,2	45,1	55
Age (median ± SD)	77,21 ± 10,40	79,2 ± 9,9	74,9 ± 14,1	77,5 ± 12,6	78 ± 14
Readmissions: 1 month (%)	7,87	9,7	6,34	11,4	11,7
Readmissions: 6 months (%)	12,59	-	15,91	29,2	20,9
Readmissions: 1 year (%)	27,3	32,6	21,43	38,4	37,5

The results of our study are consistent with other previous studies, most notably the study by Martinez-Santos^23^, which analysed all admissions of patients with a diagnosis of HF at discharge registered in Spain between January 1, 2012 and December 31, 2012. The differences found could be attributed to an older population.

The readmission rates presented in the INCA study are lower than those reported in the Omersa study^24^. This could be explained by a difference in the inclusion criteria and creation of the sample since the INCA study considered only some of the ICD-10 codes that were used in the Omersa study.

### What is already known

#### INCA score and analysis of other indicators

Several studies^25–27^ have reported predictive models for HF readmissions. Detecting patients at discharge that are most likely to be readmitted would allow for specific attention and resources to be offered that could help prevent readmission. Despite this, these predictive models are rarely used in clinical practice.

Our literature review suggests that, besides the few existing indicators, the field remains open, with no univocal process capable of stratifying patients according to risk^28^.

One of the criticisms of the studies underlying the creation of indicators is that the data often come from clinical trials, which do not represent the HF population as a whole^29^. However, for the INCA score, patients admitted for HF were included consecutively (excluding none) and thus, real-life data was provided.

The INCA score allows for an analysis of the comorbidities that most influence hospital readmission within one year in an attempt to distinguish patients who are at high risk. The expectation is that one-month readmissions can be reduced through the early implementation of appropriate follow-up approaches, both from a medical and nursing point of view^30^.

In our study, the relevant variables were the presence of previous HF without hospital admission, CKD, and COPD. These findings are consistent with previous studies^31–34^ that found an association between these comorbidities and an increased risk of readmission, suggesting that they may be reliable markers of a worse prognosis.

The predictor variables of the INCA score are consistent with the results of a recent literature review^35^ and other studies, such as the Omersa study^24^, which found that presence of MIp, neoplasia, DM, and renal failure increase the probability of hospital readmission. The Optimize-HF registry^19^ also describes a strong association between these comorbidities and a worse prognosis (though it also includes AF, worsening renal function, deterioration of respiratory function, and poor adherence to treatment and diet as precipitating factors).

#### The value of HF scores

In 2015, Spanish researchers developed the REDIN score^25^ with specific models designed to predict 30-day and 1-year readmissions. While this score includes typical HF variables, the study population was mostly men (69%) and the mean age was 66.7 years, which differs from real-world values and could negatively influence the correct application of the score.

Other scores, such as the HFPI^26^, developed by the University of Michigan, and the RR^27^, developed by the Yale New Haven Health Services Corporation/Center for Outcomes Research and Evaluation (YNHHSC/CORE), include analyses of readmissions. The former analyses readmissions over six months, while the latter analyses readmissions within one month of discharge. However, both scores analyse all-cause readmissions, whereas the INCA score only records readmissions for HF, and thus offers the opportunity to analyse a specific population.

### What this study adds

The INCA score is a simple-to-use indicator that offers healthcare personnel the opportunity to separate patients into two populations with different levels of risk, and to offer differentiated follow-up plans tailored to the control of HF and comorbidities in an attempt to reduce readmissions for HF. The most effective measures to prevent hospital readmissions will likely result from personalized follow-up based on multidisciplinary management programs, education on self-care strategies, and systematic periodic follow-ups in consultation and at home^36^.

In short, the INCA score is an indicator built on "real world" data that takes into account an elderly population with many comorbidities and adequately represents the female gender.

### Limitations and strengths

This study has the typical limitations of a retrospective study. We attempted to control for variability between the different variables through employing appropriately trained qualified personnel. The moderate number of echocardiograms and the information provided by hospital discharges led us to reject the inclusion of left ventricular systolic function in the analysis, as well as the lack of routine determination of BNP or data on the physical or cognitive abilities of the patients. which would have been of prognostic interest. Another limitation is the lack of follow-up on medication adherence, which may have contributed to patient readmission, and the lack of information on major psychiatric disorders or inflammatory diseases (in both cases, frequency or consistency of clinical history was very low).

The strengths of this study include the large number of included patients, the consecutive nature of inclusion, the ease of use of the indicator and the use of “real world” data to construct the indicator.

## Conclusion

In the first two decades of the twenty-first century, readmissions for HF have increased significantly. Among all the variables assessed, a history of HF without hospital admission, CKD, and COPD appear to have the greatest effect on readmission. Using the INCA score, comorbidities can be assessed, and patients can be separated into two groups based on readmission risk, allowing for those who require more careful assessment and follow-up to be identified.

## Data Availability

The datasets generated during and/or analyzed during the current study are available from the corresponding author on reasonable request.
